# Severe ARDS caused by adenovirus: early initiation of ECMO plus continuous renal replacement therapy

**DOI:** 10.1186/s40064-016-3571-9

**Published:** 2016-11-03

**Authors:** Sang Ook Ha, Hyoung Soo Kim, Sunghoon Park, Ki-Suck Jung, Seung Hun Jang, Sang Jin Han, Hyun-Sook Kim, Sun Hee Lee

**Affiliations:** 1 Department of Emergency Medicine, Hallym University Medical Center, Hallym University Sacred Heart Hospital, Anyang-si, Korea; 2 Thoracic and Cardiovascular Surgery, Hallym University Medical Center, Hallym University Sacred Heart Hospital, Anyang-si, Korea; 3 Division of Pulmonary, Allergy and Critical Care Medicine, Department of Internal Medicine, Hallym University Medical Center, Hallym University Sacred Heart Hospital, 22, Gwanpyeong-ro 170 beon-gil, Donan-gu, Anyang-si, Gyeonggi-do 431-070 Korea; 4 Division of Cardiology, Department of Internal Medicine, Hallym University Medical Center, Hallym University Sacred Heart Hospital, Anyang-si, Korea

**Keywords:** Adenovirus, Cidofovir, Continuous renal replacement therapy, Extracorporeal membrane oxygenation

## Abstract

The reported survival rates of patients with acute respiratory distress syndrome (ARDS) caused by human adenovirus (HAdV) pneumonia are poor. The results do not differ much in immunocompetent patients supported by extracorporeal membrane oxygenation (ECMO). We report two immunocompetent patients with severe ARDS complicating HAdV pneumonia who were treated successfully and survived to discharge. Compared with previous cases, our cases might have benefited from several factors. First, the time interval between mechanical ventilator support and ECMO implantation was shorter. Second, we implemented conservative fluid management as recommended by the ARDS network using continuous renal replacement therapy (CRRT). Third, we administered an antiviral agent as early as possible. A clinical trial of early ECMO with CRRT and the administration of cidofovir in patients with severe ARDS complicating HAdV pneumonia are needed to confirm our results.

## Background

Severe acute respiratory distress syndrome (ARDS) complicating adenovirus pneumonia has been a concern in immunocompetent adults since CDC (2001) reported two adenovirus pneumonia-related deaths in 2001. Previously, human adenovirus (HAdV) serotypes 3 and 7 accounted for the majority of severe infections; however, serotype 55 was recently identified as a pathogen of acute fatal pneumonia in immunocompetent adults (Yang et al. 2009; Kajon et al. 2010; Gu et al. 2012; Zhang et al. 2012; Cao et al. 2014). Although the mortality rate of HAdV pneumonia varies according to the study population and disease severity, a systemic review reported a mortality rate of 57.0%.

Extracorporeal membrane oxygenation (ECMO) has been used as rescue therapy for patients with severe ARDS caused by HAdV, but the outcome was not satisfactory (Gu et al. 2012; Sun et al. 2014; Prodhan et al. 2014). However, the greatest benefit of ECMO is protecting the lungs from high-pressure mechanical ventilation and oxygen toxicity, minimizing on-going lung injury. Additionally, several studies have reported that among ECMO survivors, CRRT groups had a better fluid balance compared with non-CRRT groups (Cavagnaro et al. 2007; Hoover et al. 2008; Blijdorp et al. 2009), and fluid overload at the end of ECMO was a predictor of a worse outcome (Gbadegesin et al. 2009). We believe that a low tidal volume and conservative fluid strategies are important for ARDS patients, and could be achieved by the early initiation of ECMO support and CRRT. We present two cases of severe ARDS caused by HAdV pneumonia for whom the early initiation of ECMO plus CRRT, together with an antiviral agent (cidofovir), was tried.

## Methods

The patient data were evaluated with the approval of the Institutional Review Board of the author’s hospital. Due to the purely observational, retrospective, and non-interventional nature of this study, informed consent was deemed unnecessary and was not obtained. Patient records and information were anonymized and de-identified before analysis.

Between January and May 2015, three immunocompetent adults (a 32-year-old man, 46-year-old woman, and 60-year-old man) with HAdV pneumonia were admitted to Hallym University Sacred Heart Hospital. Two of these patients were transferred to the ICU, because of severe refractory hypoxemia and our ECMO team was consulted. Severe ARDS was diagnosed using the Berlin criteria (Ferguson et al. 2012): (1) new onset of respiratory symptoms within 1 week, or new or worsening symptoms during the previous week; (2) bilateral opacities on a chest radiograph or computed tomography (CT), not fully explained by pleural effusions, lobar/lung collapse, or nodules; (3) respiratory failure not fully explained by cardiac failure or fluid overload; and (4) an arterial oxygen tension to fraction of inspired oxygen (PaO_2_/FiO_2_) ratio ≤100 mmHg on ventilator settings that include positive end-expiratory pressure (PEEP) ≥5 cm H_2_O. HAdV pneumonia was diagnosed when the following criteria were met: (1) symptoms of an acute (viral) lower respiratory tract illness; (2) newly developed lung infiltration on chest radiography or CT; and (3) detection of adenovirus DNA from sputum specimens or bronchoalveolar lavage (BAL) fluid using the polymerase chain reaction (PCR).

### ECMO and CRRT management

Two patients required veno-venous (VV) ECMO support based on the following criteria: good premorbid functioning, PaO_2_/FiO_2_ <100 on FiO_2_ 1.0 with a PEEP of >15 cm H_2_O or uncompensated hypercapnia with acidemia (pH < 7.25); and a high end-inspiratory plateau pressure (>35 cm Hg) despite application of the best standard care using a mechanical ventilator. The Centrifugal Rotaflow Pump® (Maquet, Hirrlingen, Germany) and 17–21Fr venous cannulas (DLP®; Medtronic or RMI®; Edward’s Lifesciences, Irvine, CA, USA) were used. Both common femoral veins were cannulated percutaneously using the Seldinger technique guided by fluoroscopy in the cardiac catheterization laboratory. During ECMO, nafamostat mesilate (SK Chemicals Life Science, Seoul, Korea licensed by Torii Pharmaceutical, Tokyo, Japan) was used for anticoagulation with a maintenance dose of 0.4–1.5 mg/kg/h and a target activated partial thromboplastin time (aPTT) of 60–80 s. The CRRT machine was introduced into the ECMO circuit by connecting the inlet line after the oxygenator and the outlet line before the oxygenator (Chen et al. 2014). The mechanical ventilator settings were as follows: inspiratory pressure, 10 cm H_2_O; PEEP, 10 cm H_2_O; respiration rate, 10/min; I/E ratio, 2:1; and FiO_2_, 0.21‒0.6. The target hematocrit was >30% and the platelet target >50,000‒100,000/mm^3^. Although a positive fluid balance was initially permitted up to 2–3 L/day due to the patients’ unstable hemodynamic conditions, a conservative fluid strategy, as recommended by the ARDS Clinical Trial Network, was followed thereafter (National Heart L et al. 2006; Brodie and Bacchetta 2011). Successful ECMO weaning was defined as patient survival for >24 h after ECMO removal.

## Results

### Patient 1

**Fig. 1:**
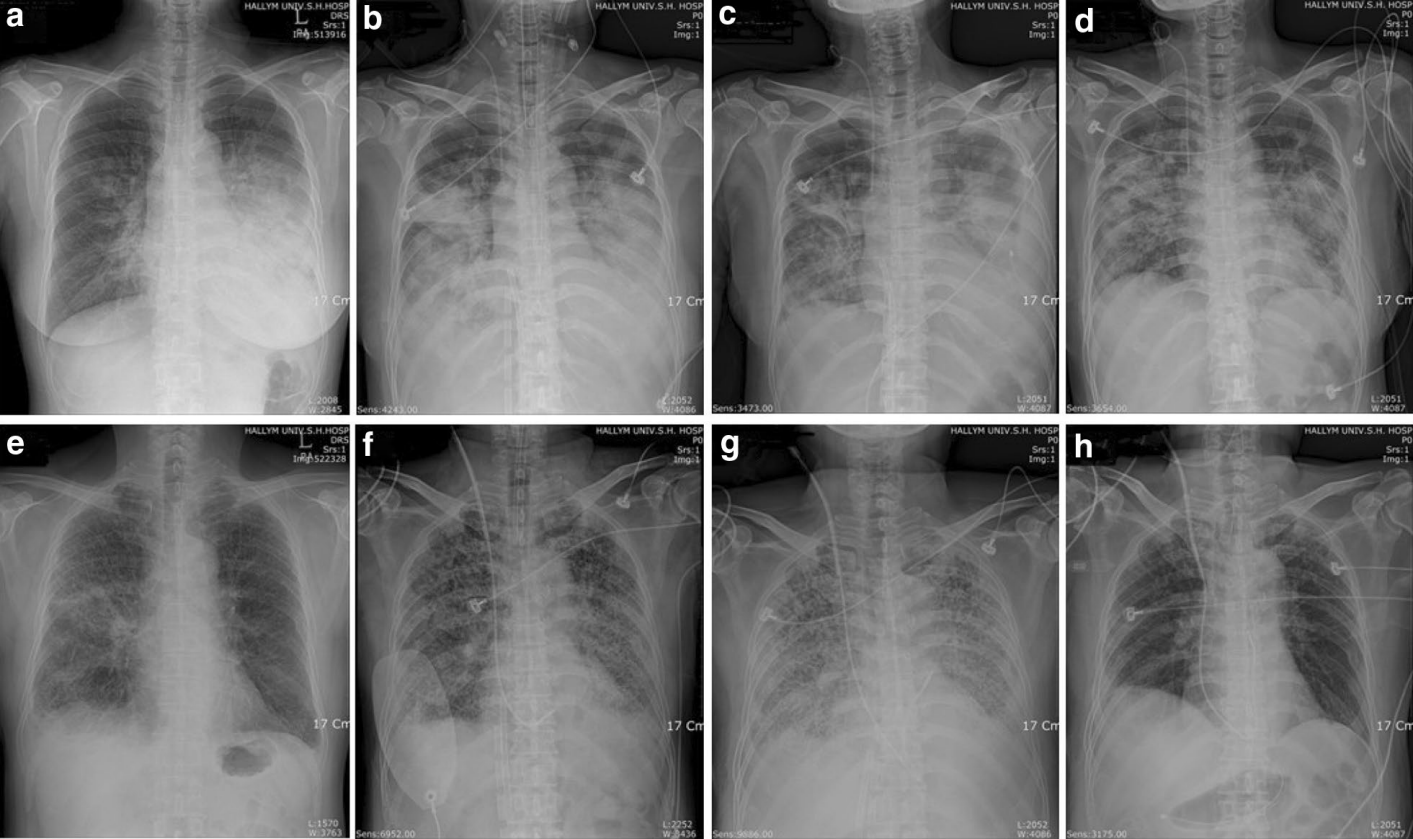
Chest radiography of two patients with severe ARDS caused by adenovirus. **a** and **e** On admission day; **b** and **f** on ECMO day 1; **c** and **g** on ECMO day 7 and; **d** and **h** on day after ECMO weaning

**Fig. 2:**
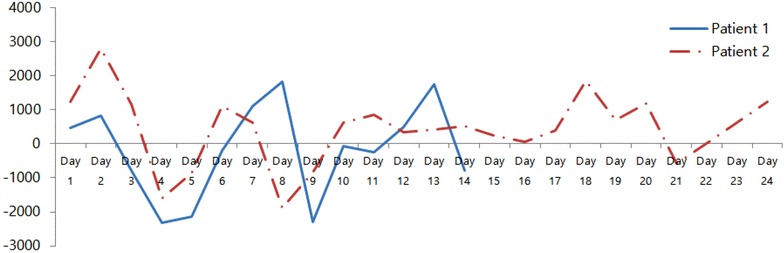
Daily net fluid balance of the two patients with severe ARDS caused by adenovirus

A 46-year-old healthy woman with a non-contributory medical history had a high fever, cough, and sputum for 5 days and was admitted to a local hospital with left lower lobe pneumonia. Despite administration of empirical antibiotic therapy comprising intravenous ceftriaxone and clarithromycin, her symptoms persisted and she developed dyspnea; at this time the patient was referred to our hospital (Fig. 1). On admission, her vital signs were systolic blood pressure 120 mm Hg, heart rate 104 beats/min, respiratory rate 24/min, and body temperature 39 °C. The white blood cell count was 11,900/mm^3^, C-reactive protein was 162.2 mg/L, and liver function tests were within normal ranges. By pulse oximetry, the initial oxygen saturation was 89% with O_2_ 8 L via facial mask. On the second day of hospitalization, the consolidation on chest X-ray was worse and her oxygen requirement markedly increased. She was transferred to the ICU and started on mechanical ventilation and a norepinephrine infusion (~0.6 μg/kg/min; Simplified Acute Physiologic Score II [SAPS II], 35). On ICU day 2, HAdV was identified by PCR in a sputum specimen obtained at the time of admission, and she was given cidofovir 5 mL/kg/week for 2 weeks. We also started intravenous methylprednisolone at 1 mg/kg q 24 h, which was later tapered slowly. However, the severe hypoxia was not improved despite a FiO_2_ of 100% and a PEEP of 15 cm H_2_O (i.e., PaO_2_/FiO_2_ = 40 mmHg), and her plateau pressure was >35 mm H_2_O. We put her in a prone position, but this did not improve her severe hypoxemia. Therefore, we started VV ECMO in the patient with concomitant CRRT. The initial ECMO settings were as follows: blood flow 4.6 L/min, sweep gas flow 6.0 L/min, and FiO_2_ 1.0 (Table 1). Her hemodynamic findings stabilized after initiating VV ECMO. We performed BAL, and the cell differential in the BAL fluid was as follows: neutrophils 45%, lymphocytes 26%, macrophages 20%, and eosinophils 9%. Microbiological testing of the BAL specimen gave a positive PCR test for HAdV, while all tests for bacteria and other viruses were negative. We continued the conservative fluid strategy throughout the ECMO treatment (Table 2) and her chest X-ray findings began to improve on ECMO day 4. She was extubated and allowed to awaken from the ECMO on day 5. On ICU day 16 (i.e., ECMO day 14), ECMO and CRRT were finally weaned and removed. Her net fluid balance was −2305 mL (−164.6 mL/day) during the ECMO period (Fig. 2). Subsequently, she was transferred to a general ward on ICU day 20 and finally discharged home. She was alive and had normal daily activities at the 1-year follow-up assessment.

**Table 1:** Clinical and hemodynamic parameters of two patients

		Pre-ECMO	ECMO day 1	ECMO day 3	ECMO day 7	ECMO day 10	ECMO day 14	Post ECMO weaning
Patient 1								
Arterial blood gas analysis	pH	7.40	7.49	7.464	7.46	7.43	7.44	7.40
	PaCO_2_, mmHg	26.8	25.8	33.7	29.7	38.5	34.1	27.9
	PaO_2_, mmHg	54.8	215.7	126.8	102.9	162.4	102.2	97.4
	SaO_2_, %	86.7	99.9	98.8	98.4	99.9	98.1	97.9
Mechanical ventilation	Ins pressure, mmHg	18	10	10	–	–	–	–
	PEEP, mmHg	15	10	10	–	–	–	–
	FiO_2_	1.0	0.3	0.3	0.3 by NC	0.3 by NC	0.3 by NC	0.3 by NC
ECMO setting	ECMO blood flow, L/min	–	4.6	4.5	4.4	3.0	2.0	–
	ECMO FiO_2_,	–	1.0	1.0	1.0	1.0	0.2	–
	Sweep gas flow, L/min	–	6.0	4.0	4	4.0	0	–
Patient 2								
Arterial blood gas analysis	pH	7.38	7.42	7.43	7.47	7.45	7.46	7.41
	PaO_2_, mmHg	33.5	30.8	32.9	35.1	40.0	32.7	38.6
	PaCO_2_, mmHg	67.6	183.8	148.4	77.3	78.5	101.5	107.2
	SaO_2_, %	92.5	99.9	99.6	96.0	96.0	98.3	97.5
Mechanical ventilation	Ins pressure, mmHg	20	10	10	10	12	10	12
	PEEP, mmHg	15	10	10	10	8	6	6
	FiO_2_	0.9	0.6	0.35	0.45	0.45	0.45	0.35
ECMO setting	ECMO blood flow, L/min	–	5	5	4.58	2.5	3.4	–
	ECMO FiO_2_,	–	1.0	1.0	1.0	1.0	1.0	–
	Sweep gas flow, L/min	–	4.5	4.5	9.5	6.5	5.8	–

*ECMO* extracorporeal membrane oxygenation, *NC* nasal cannula

**Table 2:** Hemodynamic and fluid balance parameters

	Pre-ECMO	ECMO days											
		Day 1	Day 3	Day 5	Day 7	Day 9	Day 11	Day 13	Day 15	Day 17	Day 19	Day 21	ECMO weaning day
Patient 1													
Blood pressure, mmHg	100/57	92/64	133/77	118/80	130/79	124/71	93/50	101/53	–	–	–	–	129/71
Heart rate,/min	82	87	82	90	104	99	89	91	–	–	–	–	102
Input, mL	–	3905	4135	2750	4060	3060	3340	5700	–	–	–	–	3745
Output, mL	–	3435	4908	4898	2958	5340	3090	3957	–	–	–	–	4519
CVP, cm H_2_O	17	13	16	15	8	5	1	1	–	–	–	–	0
BNP. pg/mL	152.4	117.2	656.0	187.1	84.1	60.3	37.14	22.15	–	–	–	–	44.9
Lactate, mmol/L	1.4	1.3	2.6	2.4	1.9	1.2	1.1	0.9	–	–	–	–	1.0
Patient 2													
Blood pressure, mmHg	115/78	163/95	131/77	114/63	143/84	105/68	159/95	130/74	117/69	116/71	105/62	127/84	128/102
Heart rate,/min	151	95	80	90	69	80	116	93	86	90	100	94	110
Input, mL	–	3364	5876	3125	2925	3045	3680	3505	3550	3620	3940	4045	3875
Output, mL	–	2120	4213	3992	2308	3870	2822	3099	3316	3215	3245	4633	2638
CVP, cm H_2_O	17	18	17	4	10	8	9	8	5	7	7	6	7
BNP. pg/mL	59.0	253.9	58.7	50.7	297.9	79.1	13.4	23.4	20.9	16.4	27.8	43.3	73.4
Lactate, mmol/L	3.8	2.5	1.7	1.6	2.0	1.6	1.8	1.8	1.8	1.9	2.5	2.4	2.3

*BNP* brain natriuretic peptide, *CVP* central venous pressure

### Patient 2

A 60-year-old man with a history of hypertension, variant angina, and hyperthyroidism developed high fever, cough, sputum, myalgia and dyspnea 4 days before admission. He was admitted to our hospital with a presumptive diagnosis of community-acquired pneumonia and treated with empirical antibiotic therapy comprising piperacillin–tazobactam and levofloxacin (Fig. 1). His systolic blood pressure was 100 mm Hg, heart rate 126 beats/min, respiratory rate 36/min, and body temperature 39 °C. The initial laboratory findings were as follows: WBC 14,500/mm^3^, platelets 237,000/mm^3^, CRP 302.6 mg/L, BUN 29.8 mg/dL, and creatinine 1.18 mg/dL. Liver function tests were within normal ranges. The initial O_2_ saturation was 85% and he received O_2_ (5 L/min) via facial mask. On day 3, his chest X-ray findings were worse and his oxygen requirement was increased. The patient was transferred to the ICU and treated with a non-invasive positive pressure ventilator. However, his severe hypoxemia was not improved (SaO_2_ = 94% with FiO_2_ 100% and respiratory rate = 45/min), and he was intubated. On ICU day 2, his severe hypoxemia was not improved despite mechanical ventilation (i.e., PaO_2_/FiO_2_ = 67.6 with a PEEP of 15 cm H_2_O), and his heart rate increased to 151 beats/min (SAPS II, 36). We decided to apply VV ECMO, and started CRRT for fluid management. The initial ECMO settings were as follows: blood flow 5.0 L/min, sweep gas flow 4.5 L/min, and FiO_2_ 1.0. On ECMO day 4, a PCR test for HAdV in a sputum sample taken at the time of admission was positive; based on this finding, the patient was administered cidofovir 5 mL/kg/week. The cell differential in the BAL fluid was as follows: neutrophils 43%, lymphocytes 34%, macrophage 22%, and eosinophils 1%. All tests of the BAL specimen for bacteria and viruses were negative. Although his blood gas oxygenation and chest X-ray showed an initial improvement during the early period of VV ECMO support, they were again worse on ECMO day 7 (Fig. 1). Therefore, we started treatment with methylprednisolone 2 mg/kg q 24 h, which was then tapered slowly. We attempted to maintain a conservative fluid strategy (Table 2) and the PaO_2_/FiO_2_ ratio and chest X-ray findings began to improve from ECMO day 9. On ECMO day 12, a tracheostomy was performed and ECMO and CRRT were removed on ECMO day 24. His net fluid balance was +10,219 mL (+425.8 mL/day) throughout the ECMO treatment (Fig. 2). The patient was transferred to a general ward on ICU day 32 and finally discharged home. At his 1-year follow-up assessment, the patient was ambulatory and able to perform light housework.

## Discussion

We successfully treated two patients diagnosed with adenovirus pneumonia with severe ARDS and suggest that such patients might benefit from early appropriate support using VV ECMO and CRRT.

Although no well-designed randomized controlled trials have proven its efficacy (Zapol et al. 1979; Morris et al. 1994, 2012), the conventional ventilatory support versus extracorporeal membrane oxygenation for severe adult respiratory failure (CESAR) trial and 2009 influenza A (H_1_N_1_) epidemic have promoted ECMO use worldwide (Peek et al. 2009; ANZ ECMO et al. 2009). To date, several authors have reported ECMO use in severe ARDS caused by HAdV. Sun et al. (2014) described five patients with severe ARDS with confirmed HAdV-55 infection, four of whom received VV ECMO. Low et al. (2013) reported three patients with confirmed HAdV-7 ARDS who were treated with VV ECMO; the reported survival rates were poor. Prodhan et al. (2014) also reported that only 62 (38.0%) of 163 children with adenovirus pneumonia supported using ECMO survived. However, both of our patients survived throughout the period of ECMO support. Compared with previous studies, our cases might have benefited from several factors. First, the time interval between mechanical ventilator support and ECMO implantation was shorter. Second, we followed the conservative fluid management recommended by the ARDS network, using CRRT support. Third, we administered the antiviral agent as early as possible.

The most important role of ECMO support might be ‘lung rest’ with a low tidal volume, thereby decreasing ventilator-induced lung injury (VILI). Therefore, the early initiation of ECMO seems to be an intriguing option to protect the lungs of these critically ill patients (MacLaren et al. 2012; Turner and Cheifetz 2013). In a case series in Low et al. (2013), the duration of pre-ECMO mechanical ventilation was 2, 5, and 16 days, while in Sun et al. (2014), it was 2–13 days, which were longer than in our two cases (i.e., 47 and 15 h, respectively). We also applied lung rest ventilation to the patients, as recommended by the Extracorporeal Life Support Organization (ELSO). Hence, we suggest that this early initiation of ECMO support might have played a role in our cases. However, ECMO has not been accepted as an established treatment for adults with ARDS (Morris et al. 2010; Hirshberg et al. 2013; Deutschman and Neligan 2016). Two randomized clinical trials failed to show that ECMO was efficacious in adults with ARDS (Zapol et al. 1979; Morris et al. 1994). Hence, we cannot say that ECMO treatment was directly related to patient survival in our cases (Park et al. 2010); rather, conservative fluid management using CRRT could have played a critical role (National Heart L et al. 2006). Although patients undergoing ECMO and CRRT have an increased risk of mortality compared with those receiving ECMO only, Chen et al. (2014) recently suggested that the combination of ECMO and CRRT is a safe, effective method for treating ECMO patients who have developed acute kidney disease. One advantage of CRRT is that it can improve fluid overload and electrolyte imbalance in critically ill patients. Moreover, recent studies demonstrated in porcine models that ECMO in combination with CRRT treatment could reduce inflammatory cytokines and ameliorate ECMO-induced acute kidney injury or better preserve lung parenchyma (Yimin et al. 2013; Shi et al. 2014). Our ECMO team also initiated CRRT simultaneously with ECMO support due to its immunomodulatory effect and to improve the fluid/electrolyte/acid–base balance. Although Wolf et al. (2013) were concerned about precipitating intravascular depletion by CRRT at a time of maximal capillary leakage, we permitted, if needed, a positive fluid balance of up to 2–3 L in our cases on ECMO days 1–2. However, the total net fluid balance of the two cases during the entire ECMO period was −164.6 to 425.8 mL/day, respectively.

Regarding the treatment of severe HAdV pneumonia, no controlled trials have demonstrated the effectiveness of antiviral agents. Recently, Kim et al. (2015) reported that six patients with severe HAdV pneumonia who had received cidofovir a median of 7.1 days after symptom onset recovered to discharge. They suggested that the early administration of cidofovir is an important treatment option, especially in cases of HAdV-55 infection. In previous reports, however, many cases with HAdV infections did not receive cidofovir, and the few patients who received the agent had poor outcomes. In our cases, the time interval from symptom onset to cidofovir administration was 8–9 days, respectively. However, the clinical, radiological, and laboratory findings of our patients improved gradually. The only adverse reaction was a skin rash in one patient. The effectiveness of cidofovir for severe HAdV pneumonia should be investigated further.

There were several limitations to this study. First, only two patients were reviewed due to the rarity of patients with HAdV pneumonia who receive ECMO support. Therefore, more experience is needed to draw a firm conclusion regarding the standard therapy. Second, the HAdV serotypes were not identified in our patients; therefore, the clinical efficacy of cidofovir for pneumonia caused by HAdV of specific serotypes could not be determined. Despite these limitations, however, we suggest that to reduce VILI and extravascular lung water, early initiation of concomitant VV ECMO and CRRT support should be considered in patients with severe ARDS complicating HAdV pneumonia.

## Conclusions

We report two patients with severe ARDS complicating HAdV pneumonia who were treated successfully and survived to discharge. We suggest that this patient group might benefit from early appropriate support with VV ECMO plus CRRT and cidofovir administration. Further clinical analysis of this treatment strategy is needed to confirm our results.

## Abbreviations

ARDS: acute respiratory distress syndrome
BAL: bronchoalveolar lavage
CESAR: conventional ventilatory support versus extracorporeal membrane oxygenation for severe adult respiratory failure
CRRT: continuous renal replacement therapy
CT: computed tomography
ECMO: extracorporeal membrane oxygenation
ELSO: Extracorporeal Life Support Organization
HAdVs: human adenovirus
ICU: intensive care unit
PEEP: positive end expiratory pressure
PCR: polymerase chain reaction
SAPS: simplified acute physiology score
VILI: ventilator-induced lung injury
VV: veno-venous
